# Effects of the poultry red mite (*Dermanyssus gallinae*) load on the plumage condition in commercial laying hen farms

**DOI:** 10.1371/journal.pone.0277513

**Published:** 2022-11-11

**Authors:** Ruben Schreiter, Marion Herzog, Markus Freick

**Affiliations:** 1 ZAFT e.V., Centre for Applied Research and Technology, Dresden, Germany; 2 HTW Dresden – University of Applied Sciences, Dresden, Germany; University of California Davis School of Veterinary Medicine, UNITED STATES

## Abstract

Plumage damage (PD) resulting from severe feather pecking (SFP) is a significant problem for animal welfare, performance, and economics in commercial laying hen farms. Genetics, nutrition, and housing conditions are central complexes that contribute to the multifactorial cause of SFP. Practical experience suggests that infestation by the poultry red mite (PRM), which is the most severe ectoparasite of laying hens in cage-free housing systems, may be a risk factor for the occurrence of PD, although evidence-based findings are lacking. The objective of this longitudinal observational field study was to investigate the effects of PRM infestation of commercial laying flocks on the occurrence of PD. Integument scoring (plumage damage and skin lesions) and the quantification of PRM infestation using mite traps were conducted during the laying period of 28 laying flocks, with an average flock size of 12,357 birds in barn (n = 21) or free-range (n = 7) systems. Across all flocks and survey times, the median PRM mass per trap was 0.7 mg (1.-3. quartile: 0.0–19.3 mg/trap), corresponding to a median count of 65.2 mites/trap (1.-3. quartile: 0.0–246.8 mites/trap). Binary logistic regression models revealed an association between PD and skin lesions with hen age, housing system, and hybrid type (p<0.001). The PRM load also affected the plumage condition, where PD increased with increasing PRM infestation (p<0.001). In addition, the PRM load tended to have an effect on skin injuries (p = 0.097). In conclusion, this longitudinal study identified the PRM load in laying hen flocks as a risk factor for PD.

## Introduction

Poultry red mite (PRM; Dermanyssus gallinae) is the most severe ectoparasite of laying hens in cage-free housing systems worldwide, as infestations are difficult to control [1]. The parasite resides mainly in cracks and hiding places during the day and consumes blood meals from the hens at night, which are necessary for completion of its developmental stages [2]. Blood sucking by mites can lead to restlessness, reduced laying performance and egg quality, anemia, and increased mortality, especially under heavy infestations [3].

Severe feather pecking (SFP) and cannibalism are the most significant behavioural disorders in laying hens; they occur in all housing systems and cause serious problems for animal welfare, biological performance, and the economic success of egg production [4, 5]. SFP results in plumage damage (PD) is not an aggressively motivated behavior [6]. The risk of SFP is influenced by a variety of factors related to genetics, feeding, housing, and management [7–9]. Practical experience also suggests a relationship between PRM infestation and SFP [2, 10]. A cross-sectional study by Heerkens et al. [11] observed lower PD in flocks without PRM compared to those with PRM infestations. Recent studies that included direct behavioral observations showed a decrease in SFP due to a treatment-induced reduction in PRM infestation in laying flocks [12, 13]. A more practical approach to the assessment of behavioural disorders that can be implemented in farm routines is integument scoring [14], which is an indirect method used to quantify SPF and cannibalism [15].

Yet, the association of PRM exposure and the occurrence of PD and skin lesions is reported only anecdotically [2, 10, 11]. In the absence of longitudinal studies on the indirect quantification of SFP and cannibalism by integument scoring and its association with PRM infestation, the objective of this study was to investigate the effects of PRM load on PD and skin injuries using a longitudinal design. We hypothesized that PRM exposure over the laying period would be associated with 1) the occurrence of PD and 2) skin lesions in commercial laying flocks.

## Materials and methods

### Flocks and study design

Animals from 28 flocks (n = 9 white-egg laying (WL) flocks and n = 19 brown-egg laying (BL) flocks) on 11 laying hen farms in Saxony/Germany were included in this field study. The hens were kept in barns with aviaries (n = 21 flocks) or free-range systems (n = 7 flocks; non-organic, 4 m^2^ free-range area per hen, barn with aviary systems) with a median flock size of 12,357 hens (1.-3. quartile: 5,671–16,996) according to the legal requirements of the EU and Germany. The study was reviewed by the Country Directorate of Saxony/Germany as the responsible animal ethics committee and not classified as animal experiment (reference DD24.1-5131/277/42).

The pullets were housed in the laying houses at 17.4 ± 0.8 weeks of life (LW). To assess the integument condition and to quantify mite infestation in the flocks, we used an observational longitudinal study design. The first surveys were carried out one month after housing, followed by a monthly visit to the flock. Thus, data from the first to the eleventh laying month (LM) are available. The visits took place in the following weeks of life (LW; mean ± SD): 23.3 ± 1.5 LW, 28.1 ± 1.5 LW, 32.5 ± 1.7 LW, 36.7 ± 1.6 LW, 41.2 ± 1.3 LW, 45,3 ± 1.8 LW, 50.1 ± 2.2 LW, 54.8 ± 1.7 LW, 58.8 ± 2.0 LW, 62,3 ± 2.4 LW, and 66.1 ± 2.7 LW.

### Integument scoring

For the indirect determination of the occurrence of SFP and cannibalism, a scoring of the integument was performed on predetermined dates (LM 1, 2, 3, 5, 7, 9, and 11). For this, individual birds were regarded as experimental units. The sample size (50 hens per time point and flock) and the random selection of animals in the flock from all housing areas and levels were carried out according to Kaesberg et al. [16].

Integument scoring for plumage loss and skin injuries was performed by the same observer according to the Welfare Quality^®^ [14] guidelines. Intra-observer reliability was assessed at three time points during the study, with 50 hens each time. Both traits were divided into three scores (plumage: 0 = intact plumage, 1 = moderate plumage damage with one or more featherless areas ≤5 cm, 2 = severe plumage damage with one or more featherless areas >5 cm; skin lesions: 0 = intact skin, 1 = moderate skin lesions ≤1 cm, 2 = severe skin lesions > 1 cm). Plumage was scored in three body regions, i.e., the dorsal neck, back, and belly. In addition to the three individual scores for the plumage regions, a total plumage score was calculated for each animal by adding the individual scores [17]. For each of the assessed traits, 9,800 data sets were recorded.

### Quantification of the mite load

We monitored the PRM using mite traps to quantify the PRM infestation. For this, AviVet Traps (AviVet B.V., Lunteren/NL) were used, which were previously validated as a suitable tool for the detection of eggs, larvae, nymph stages, and adult mites on laying hen farms [18]. During all flock visits, ten AviVet traps per flock were placed in the laying houses for the optimal period of 48 hours [19]. For this purpose, ten locations were defined in each study flock prior to the first placement of the mite traps. These locations were selected along typical routes from the hiding place of the mites during the day to the resting place of the hens at night (3 aviary racks, 3 perches, 2 watering lines, and 2 nest areas), where the AviVet traps were fixed using the included cable tie [18]. When the traps were removed, each trap was packed in a plastic bag (AviVet B.V., Lunteren/NL). All of the individually sealed bags were placed in another plastic bag and stored in a freezer (Premium NoFrost, Liebherr-Hausgeräte GmbH, Ochsenhausen/DE) for at least 48 hours at -20°C [20].

Further analysis of the AviVet traps was performed in the laboratory to determine the PRM mass following Lammers et al. [18]. The mite traps were removed from the plastic bag after thawing for 14 h at 12 °C, and the PRMs in the bag were poured into a weighing dish (disposable weighing dish, neoLab Migge GmbH, Heidelberg/DE). Subsequently, the corrugated cardboard was pressed out of the plastic tube using a stainless steel micropistil (neoLab Migge GmbH, Heidelberg/DE). All of the mite stages present were wiped off from the opened corrugated cardboard and plastic tube using a disposable brush (Outus, Shanghai/CHN) and added to the mites from the bag in the weighing dish. Afterwards, weighing was performed with a SI-234 precision balance (Denver Instrument GmbH, Göttingen/DE).

A total of 3,080 mite trap data sets were available.

### Statistical analyses

Microsoft Excel^®^ (version 2013, Microsoft Corporation, Redmond/USA) was used for data collection and processing and for creating selected diagrams. SAS 9.4 for Windows (SAS Institute Inc., Cary, NC, USA) and IBM SPSS Statistics (Version 23, SPSS Inc., Chicago/USA) were used to analyze the data.

A concordance analysis was performed to quantify the degree of agreement in the integument scores. For this purpose, the prevalence-adjusted and bias-adjusted kappa (PABAK) values were calculated as characteristics of the intra-observer reliability according to Gunnarsson et al. [21]. PABAK values were interpreted as follows [22, 23]: <0.20 = insufficient, 0.21–0.40 = sufficient, 0.41–0.60 = moderate, 0.61–0.80 = good, and >0.80 = very good degree of agreement.

The number of PRM stages per trap was calculated from the determined PRM mass (number of mites = 58.5 + 9.56 * mite mass (mg), [18]), and the mean mite mass per trap was determined as the arithmetic mean of the ten analyzed mite traps per farm visit.

For the univariate analyses of the effects of PRM load on plumage condition and skin lesions, quartiles were calculated for the mean mite mass per trap using data from the entire study period. Flocks were classified on each monitoring date as follows: PRM/(low mite load, i.e., flocks with mite mass <1st quartile), PRM + (moderate mite load, i.e., flocks with mite mass between the 1st and 3rd quartile), or PRM ++ (high mite load, i.e., flocks with mite mass >3rd quartile). A comparison of total plumage scores and skin scores between the three classes in each LM was performed using a Kruskal–Wallis test [24]. The Benjamini–Hochberg procedure was used to control for false discovery rates due to multiple testing [25].

Binary logistic regression (BLR) models were used with the total plumage score and skin lesions as dependent variables [26]. The age of the hens [17], hybrid type, and housing system [27] are known risk factors associated with integument condition and were added into the models. Additionally, the mean mite mass per trap was included in the models to test the study hypotheses. Farm was treated as a random effect and therefore characterized the farm-specific management factors to which the herds were subjected. Multiple logistic regression models, rather than ordinal, were used because some values had very few observations. Since only few animals showed severe feather damage we decided to dichotomize plumage score and skin lesion instead of using the raw values to achieve more power to identify risk factors. Therefore, the ordinal data scaling (defined by Welfare Quality^®^ [14]) was converted to nominal scaling (total plumage scores: 0 for scores 0, 1, and 2 and 1 for scores ≥3; skin lesions: 0 for score 0 and 1 for scores ≥1). The absence of multicollinearity was ensured by calculating the Pearson correlation coefficients and using collinearity diagnostics with the variance inflation factor and condition index [28, 29]. Since we did not find any multicollinearity in the data, we assume that the effects examined can be considered independent risk factors with p≤0.05.

Differences were considered statistically significant for p-values of ≤0.05 and tended to be significant if 0.05<p≤0.1.

## Results

The PABAK values of 0.90 for neck plumage, 0.94 for back plumage, 0.91 for belly plumage, and 0.93 for skin lesions indicated very good intra-observer reliabilities.

**Fig 1:**
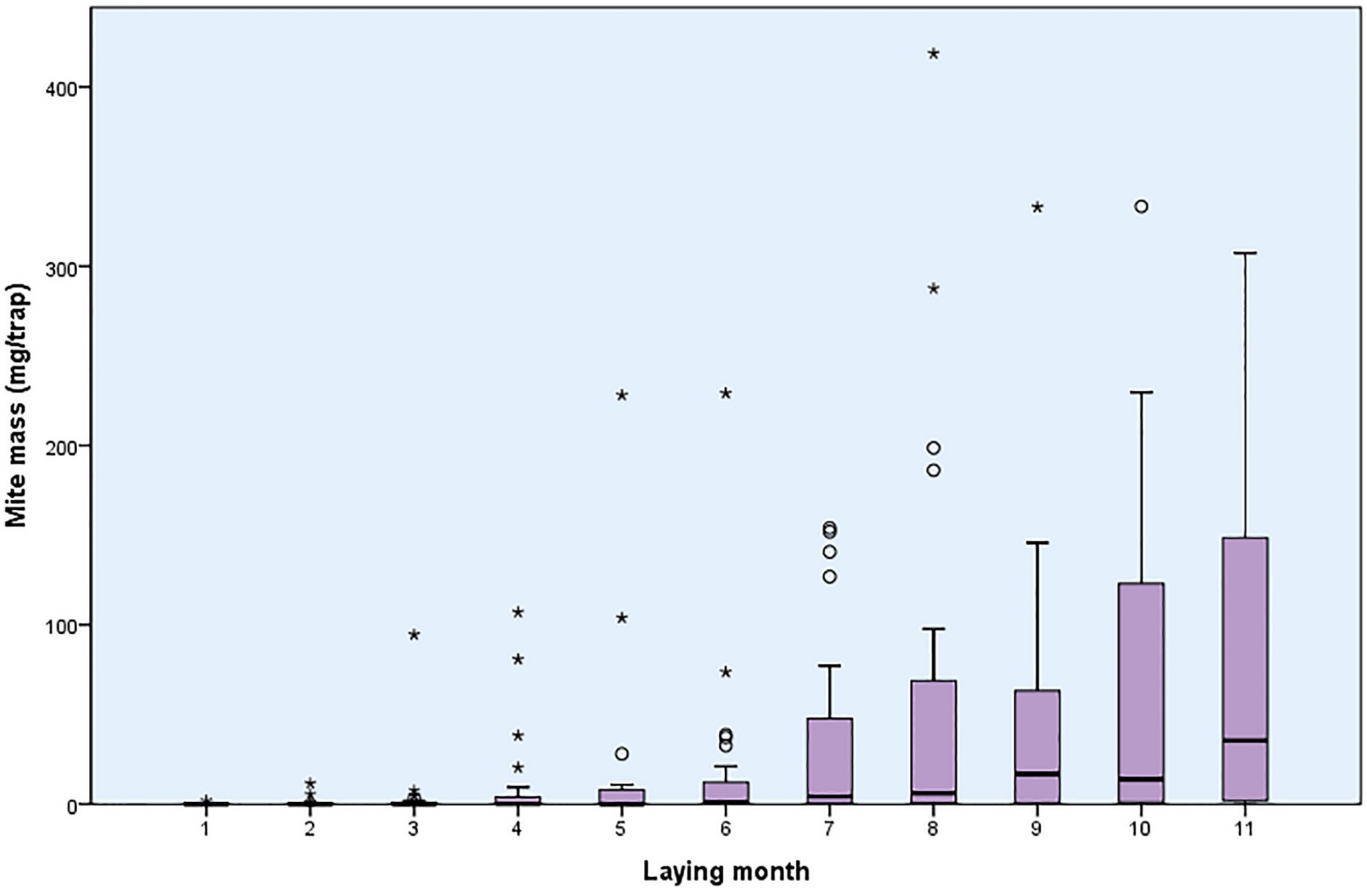
Poultry red mite load detected by AviVet traps (AviVet B.V., Lunteren/NL) in the investigated laying hen flocks during the laying period.

PRM infestations were found in 25/28 flocks (89.2%) during the laying period. Considering all monitoring dates and flocks, the median mite mass per trap was 0.7 mg (1.-3. quartile: 0.0–19.3 mg/trap), resulting in a median number of 65.2 mites/trap (1.-3. quartile: 0.0–246.8 mites/trap). Fig 1 shows the PRM infestation during the laying period represented as the mean mite mass per trap in the flocks.

**Fig 2:**
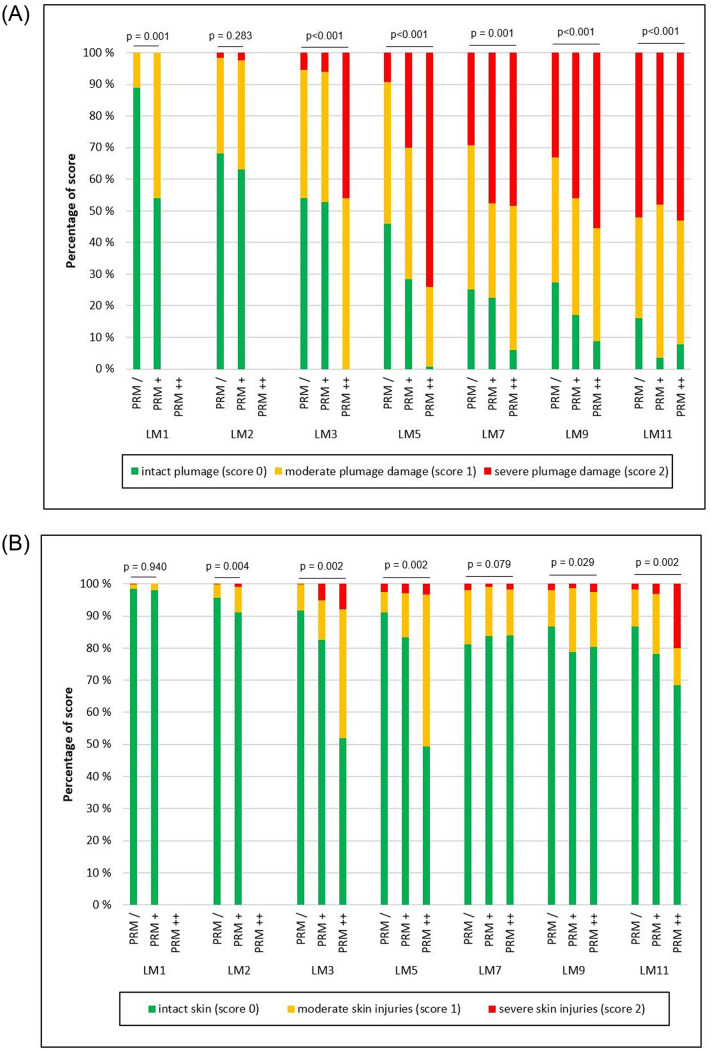
Effect of poultry red mite load at the time of scoring on plumage condition (A) and skin injuries (B) during the laying period. PRM–poultry red mite; LM–laying month. Quartiles were calculated for the mean mite mass (of 10 mite traps) per flock and monitoring date using data from the entire study period: Flocks were classified for each monitoring date as follows: PRM / (low mite load, i.e. flocks with mean mite mass <1. quartile), PRM + (moderate mite load, i.e. flocks with mite mass between 1.- 3. quartile), and PRM ++ (high mite load, i.e. flocks with mite mass >3. quartile). The percentage shown per plumage score corresponds to the arithmetic mean of the three assessed plumage regions (dorsal neck, back, and belly plumage).

The plumage condition and skin injuries over the laying period for the three PRM load categories are shown in Fig 2; differences in the plumage condition were found between the different PRM groups in all LMs (p<0.001), with the exception of LM 2. The PRM load also tended to have an effect on the skin lesions in all LMs apart from LM 1 and 7.

BLR models were used to analyze the effect of the hens’ age, hybrid type, housing system, and PRM load on the occurrence of plumage damage and skin lesions (Table 1). Plumage damage and skin lesions increased with age (p<0.001). The hybrid type was associated with a less damaged plumage condition in WL flocks (p<0.001). The PRM mass showed an association with the plumage condition, where a higher prevalence of plumage damage was observed with an increasing PRM load (p<0.001). The PRM load also had a tendencial effect on the occurrence of skin injuries (p = 0.097).

**Table 1:** Effects of age, hybrid type, housing system and poulty red mite load on the occurrence of plumage damage and skin lesions in laying hen farms: Results of logistic regression models with farm as random effect. Plumage damage was associated significantly with age, hybrid type, housing system, and mite load. Skin injuries were associated significantly with age and housing system.

Trait	Coefficients (SE)	Odds ratio (95% CI)	p-value
**Total plumage score** ^1^			
Age	0.11 (0.02)	1.12 (1.11–1.13)	<0.001
Hybrid-type^a^	-0.45 (0.12)	0.64 (0.50–0.81)	<0.001
Housing system^b^	4.64 (0.24)	104.40 (64.04–170.20)	<0.001
Mite mass (mg)	0.01 (0.00)	1.01 (1.01–1.01)	<0.001
Intercept	-10.66 (0.77)		
**Skin injuries**			
Age	0.03 (0.01)	1.03 (1.03–1.04)	<0.001
Hybrid-type*	-0.15 (0.10)	0.83 (0.58–1.05)	0.130
Housing system**	2.29 (0.24)	9.91 (6.25–15.70)	<0.001
Mite mass (mg)	0.004 (0.001)	1.000 (1.000–1.001)	0.097
Intercept	-5.57 (0.43)		

SE–standard error, CI–confidence interval

^a^brown-egg layers as reference

^b^free-ranging systems as reference

^1^Plumage was scored in three body regions, i.e., the dorsal neck, back, and belly. A total plumage score was calculated for each animal by adding the individual scores.

## Discussion

To identify possible effects of PRM infestation on PD and skin injuries, the study investigated the integument condition and the presence of PRM in laying hen flocks over the laying period in a longitudinal study design. The AVIVET mite traps validated by Lammers et al. [18] were suitable for mite monitoring.

In this study, PRM was detected in 89% of the laying flocks. Thus, the detection rate was in accordance with data from previous studies. An epidemiological report [30], observed a PRM prevalence of 94% in Germany and 83% across eleven European countries. For alternative housing systems, Sparagano et al. [1] reported prevalences that differed significantly between countries, ranging from 33% (UK) to 83% (NL). Due to the much shorter intervals of service periods and fewer hiding places, the prevalence of PRM is considerably lower in broiler farms [1]. The high detection rate of PRM underlines the high importance of this ectoparasite in laying hen farming, which has gained additional impact in Europe due to the change from cage to alternative housing systems or enriched cages [31]. The observed increase in the PRM load during the laying period was in accordance with previous studies [3].

Several risk factors were identified for plumage loss and skin injuries as indirect traits to quantify SFP and cannibalistic pecking in laying flocks. The observed increase in PD with increasing hen age, in barn versus free-range systems, and in brown- versus white-egg layers is consistent with previous field studies [11, 27]. Given the higher PD with increasing PRM load, mite infestation can be considered an additional risk factor for SFP. It is likely that the PD resulted primarily from SFP, particularly in the scored body regions [15]. To the authors’ knowledge, this is the first time that the effect of the PRM load on the plumage condition has been demonstrated in a longitudinal study design over the laying period. Recently, Petersen et al. [13] and Temple et al. [12], using direct behavioral observations, found a reduction in SFP with treatment-induced decrease in the PRM load in laying flocks treated with fluralaner, which is an isoxazoline to be administered via the drinking water for mite control. Temple et al. [12] discussed the concomitant reduction in pain and frustration with the reduction in the PRM load in the flocks as relevant to their findings. In practical observations, skin irritation and increased stress as a result of PRM infestation have also been hypothesized as possible causes of the negative effects on SFP [2, 10]. Heerkens et al. [11] found lower PD in flocks without PRMs compared to moderate or severe PRM infestations in laying hens scored once at 60 weeks of age.

On skin injuries as an indirect trait for cannibalistic pecking, the study showed a tendencial effect with a higher prevalence of skin lesions with increasing mite infestation. Although SFP and cannibalism are two different behavioral disorders, they are based on similar complexes of causes [8]. In this respect, the possible causes for the associations of SFP and mite infestation can also be applied to the association between PRM and cannibalism.

In this study, an increased risk of PD and skin lesions was added to the known effects of PRM infestation in laying flocks. Given the adverse effects on animal health and performance [3], the economic losses due to PRM infestation of EUR 0.50 to 1.00 per hen and year [10], and the animal welfare issues related to integument condition, effective strategies to control PRM infestations in commercial flocks are required. For egg producers, the adverse effects of PRM load on the performance are also of high interest. PRM infestation is accompanied by a reduction of laying performance and egg weights [12]. Due to the particular lifestyle of PRMs, with includes long stays away from their hosts in hiding places that are difficult to access [1], alternative strategies, such as the heat treatment of barns [32] or medical treatment of hens via drinking water [12, 13], are becoming increasingly important. Decru et al. [33] recommended an integrated pest management approach to PRM control, with the prevention of introduction and the monitoring of parasite loads as key approaches.

## Conclusions

Given the negative effects of PRM infestation on the plumage condition of laying hens identified in this study, the control of the PRM population should be considered an important component in reducing SFP and cannibalism in laying flocks. Egg producers should conduct targeted monitoring and, if indicated, control of PRM in addition to optimizing other factors to prevent PD.

## References

[1] Sparagano O., Pavlicevic A., Murano T., Camarda A., Sahibi H., Kilpinen O., et al. 2009. Prevalence and key figures for the poultry red mite *Dermanyssus gallinae infections* in poultry farm systems. Experimental and Applied Acarology 48, 3–10. 19160060 10.1007/s10493-008-9233-z

[2] Mul M., van Niekerk T., Chirico J., Maurer V., Kilpinen O., and Sparagano O. 2009. Control methods for *Dermanyssus gallinae* in systems for laying hens: results of an international seminar. World Poultry Science Journal 6, 589–600.

[3] Pavlicevic A., Pavlovic I., Ratajac R., Popovic D., Davidovic B., and Krnjajic D. 2019. Poultry welfare in terms of poultry red mite (*Dermanyssus gallinae*) impact and control. Biotechnology in Animal Husbandry 35. doi: 10.2298/BAH1901001P

[4] Sherwin C. M., Richards G.J., and Nicol C.J. 2010. Comparison of the welfare of layer hens in 4 housing systems in the UK. British Poultry Science 51, 488–499. doi: 10.1080/00071668.2010.502518 20924842

[5] Liebers C. J., Schwarzer A., Erhard E., Schmidt P., and Louton H. 2019. The influence of environmental enrichment and stocking density on the plumage and health conditions of laying hen pullets. Poultry Science 98, 2474–2488. doi: 10.3382/ps/pez024 30715510 PMC6527515

[6] Savory C.J. 1995. Feather pecking and cannibalism. Worlds Poultry Science Journal 51, 215–219.

[7] Van Krimpen M., Kwakkel R., Reuvekamp B., van der Peet-Schwering C., den Hartog L., and Verstegen M. 2005. Impact of feeding management on feather pecking in laying hens. World’s Poultry Science Journal 61, 663–686.

[8] Janczak A. M., and Riber A. B., 2015. Review of rearing-related factors affecting the welfare of laying hens. Poultry Science 94, 1454–1469. doi: 10.3382/ps/pev123 26009752 PMC4991062

[9] Preisinger R. 2021: Commercial layer breeding: Review and forecast. Züchtungskunde 93, 210–228.

[10] Mozafar, F. 2013. Poultry red mite—a major challenge for egg producers. Poultry News. https://lohmann-breeders.com/media/poultrynews/de/PN-2013-02-DE.pdf (accessed on 1 July 2022).

[11] Heerkens J.L.T., Delezie E., Kempen I., Zoons J., Ampe B., Rodenburg T.B., et al. 2015. Specific characteristics of the aviary housing system affect plumage condition, mortality and production in laying hens. Poultry Science 94, 2008–2017. doi: 10.3382/ps/pev187 26188031

[12] Temple D., Manteca X., Escribano D., Salas M., Mainau E., and Zschiesche E. 2020. Assessment of laying-bird welfare following acaricidal treatment of a commercial flock naturally infested with the poultry red mite (*Dermanyssus gallinae*). Plos One 15. doi: 10.1371/journal.pone.0241608 33211741 PMC7676655

[13] Petersen I., Johannhörster K., Pagot E., Escribano D., Zschiesche E., Temple D. et al. 2021. Assessment of fluralaner as a treatment in controlling *Dermanyssus gallinae* infestation on commercial layer farms and the potential for resulting benefits of improved bird welfare and productivity. Parasites Vectors 14, 181. doi: 10.1186/s13071-021-04685-7 33789728 PMC8011190

[14] Welfare Quality^®^. 2009. Welfare Quality^®^ assessment protocol for poultry (broilers, laying hens). Welfare Quality^®^ Consortium, Lelystad, the Netherlands.

[15] Bilcik B., and Keeling L. 1999. Changes in feather condition in relation to feather pecking and aggressive behaviour in laying hens. British Poultry Science 40:444–451. doi: 10.1080/00071669987188 10579400

[16] Kaesberg A.-K. U., Louton H., Erhard M., Schmidt P., Zepp M., Helmer F., et al. 2018. Development of a prognostic tool for the occurrence of feather pecking and cannibalism in laying hens. Poultry Science 97, 820–833. doi: 10.3382/ps/pex369 29294110

[17] Schreiter R., Damme K., Klunker M., Raoult C., von Borell E., and Freick M. 2020: Effects of edible environmental enrichments during the rearing and laying periods in a littered aviary–Part 1: Integument condition in pullets and laying hens. Poultry Science 99, 5184–5196. doi: 10.1016/j.psj.2020.07.013 33142434 PMC7647713

[18] Lammers G.A., Bronneberg R.G.G., Vernooij J.C.M., and Stegeman J.A. 2016. Experimental validation of the Avivet Trap, a toll to quantitatively monitor the dynamics of *Dermanyssus gallinae* populations in laying hens. Poultry Science 96, 1563–1572. doi: 10.3382/ps/pew428 27920194

[19] Nordenfors H., and Chirico J. 2001. Evaluation of a sampling trap for *Dermanyssus gallinae* (Acari: Dermanyssidae). Journal of Economic Entomology 94, 1617–1621. 11777073 10.1603/0022-0493-94.6.1617

[20] Nordenfors H., and Höglund J. 2000. Long term dynamics of Dermanyssus gallinae in relation to mite control measures in aviary systems for layers. British Poultry Science 41, 533–540. doi: 10.1080/713654991 11201430

[21] Gunnarsson, S., Algers, B., and J. Svedberg. 2000. Description and evaluation of a scoring system of clinical health in laying hens. In Gunnarsson, S. Laying hens in loose housing systems. Doctoral thesis. Swedish University of Agricultural Sciences, Uppsala.

[22] Landis J. R., and Koch G.G. 1977. The Measurement of Observer Agreement for Categorical Data. Biometrics, 33, 159 843571

[23] Kwiecien R., Kopp-Schneider A., and Blettner M. 2011. Concordance analysis–part 16 of a series on evaluation of scientific publications. Deutsches Ärzteblatt International 2011, 108, 515–521. doi: 10.3238/arztebl.2011.0515 21904584 PMC3165924

[24] Du Prel J.-B., Röhrig B., Hommel G., and Blettner M. 2010. Selection of statistical test methods. Deutsches Ärzteblatt International 107:343–348. 20532129 10.3238/arztebl.2010.0343 PMC2881615

[25] Victor A., Elsäßer A., Hommel G., and Blettner M. 2010. Judging a Plethora of p-values: How to contend with the problem of multiple testing. Part 10 of a series on evaluation of scientific publications. Deutsches Ärzteblatt International 107:50–56. 20165700 10.3238/arztebl.2009.0050 PMC2822959

[26] Baltes-Götz, B. 2000. Logistische Regressionsanalyse mit SPSS. Accessed May 2022. https://www.uni-trier.de/fileadmin/urt/doku/logist/logist.pdf

[27] Spindler B., Giersberg M. F., Andersson R., and Kemper N. 2016. Keeping laying hens with untrimmed beaks–A Review of the status quo in practice and science. Züchtungskunde 88:475–493.

[28] Menard S. 1995. Applied Logistic Regression Analysis. 1st ed. Sage, Thousand Oaks, California.

[29] Field A. 2013: Discovering Statistics Using IBM SPSS Statistics. 4th rev. ed. Sage, Thousand Oaks, California.

[30] Mul, M. 2013. Fact sheet: The Poultry Red Mite, *Dermanyssus gallinae*–A small pest that packs a big punch 2013. Accessed May 2022. https://www.researchgate.net/publication/258553789_Fact_sheet_Poultry_Red_Mite_in_Europe.

[31] Sigognault Flochlay A., Thomas E. and Sparagano O. 2017: Poultry red mite (*Dermanyssus gallinae*) infestation: a broad impact parasitological disease that still remains a significant challenge for the egg-laying industry in Europe. Parasites & Vectors 10, 357. doi: 10.1186/s13071-017-2292-4 28760144 PMC5537931

[32] Mul M.F., van Vugt S.M.A., Goselink Y.S.M., and van den Brand H. 2020. Effects of heating laying hen houses between consecutive laying cycles on the survival of the poultry red mite Dermanyssus gallinae. Veterinary Parasitology 288, 109307. doi: 10.1016/j.vetpar.2020.109307 33220641

[33] Decru E., Mul M., Nisbet A.J., Vargas Navarro A.H., Chiron G., Walton J., et al. 2020. Possibilities for IPM Strategies in European Laying Hen Farms for Improved Control of the Poultry Red Mite (Dermanyssus gallinae): Details and State of Affairs. Frontiers in Veterinary Science 7, 565866. doi: 10.3389/fvets.2020.565866 33282928 PMC7705068

